# Probenecid Blocks Human P2X7 Receptor-Induced Dye Uptake via a Pannexin-1 Independent Mechanism

**DOI:** 10.1371/journal.pone.0093058

**Published:** 2014-03-26

**Authors:** Archana Bhaskaracharya, Phuong Dao-Ung, Iman Jalilian, Mari Spildrejorde, Kristen K. Skarratt, Stephen J. Fuller, Ronald Sluyter, Leanne Stokes

**Affiliations:** 1 Sydney Medical School Nepean, University of Sydney, Nepean Hospital, Penrith, New South Wales, Australia; 2 School of Biological Sciences, Illawarra Health and Medical Research Institute, University of Wollongong, Wollongong, New South Wales, Australia; 3 Health Innovations Research Institute, School of Medical Sciences, RMIT University, Bundoora, Melbourne, Victoria, Australia; Dalhousie University, Canada

## Abstract

P2X7 is a ligand-gated ion channel which is activated by ATP and displays secondary permeability characteristics. The mechanism of development of the secondary permeability pathway is currently unclear, although a role for the hemichannel protein pannexin-1 has been suggested. In this study we investigated the role of pannexin-1 in P2X7-induced dye uptake and ATP-induced IL-1β secretion from human monocytes. We found no pharmacological evidence for involvement of pannexin-1 in P2X7-mediated dye uptake in transfected HEK-293 cells with no inhibition seen for carbenoxolone and the pannexin-1 mimetic inhibitory peptide, ^10^Panx1. However, we found that probenecid inhibited P2X7-induced cationic and anionic dye uptake in stably transfected human P2X7 HEK-293 cells. An IC_50_ value of 203 μM was calculated for blockade of ATP-induced responses at human P2X7. Probenecid also reduced dye uptake and IL-1β secretion from human CD14^+^ monocytes whereas carbenoxolone and ^10^Panx1 showed no inhibitory effect. Patch clamp and calcium indicator experiments revealed that probenecid directly blocks the human P2X7 receptor.

## Introduction

The P2X7 receptor (P2X7) is a ligand-gated ion channel activated by extracellular ATP [1]. P2X7 activation opens an ion channel pore allowing permeation of mono- and divalent cations such as Na^+^, K^+^ and Ca^2+^. During sustained activation over a timescale of seconds, the uptake of large organic cations (and anions) can be measured, a feature known as secondary permeability (reviewed in [2]). At least two distinct pathways are thought to exist for uptake of cations and anions into macrophages [3]. Whilst it is still not known what the physiological role of the secondary pore pathway actually is, it is clear that signals mediated through this pathway play an important role in P2X7 downstream signalling. P2X7 is an important regulator of pro-inflammatory IL-1β and interleukin 18 (IL-18) cytokine secretion from monocytes, macrophages and microglia [4]. A mutation in the human P2X7 receptor C-terminus which abolishes the secondary pore pathway also impairs induction of IL-1β and IL-18 processing and secretion [5], [6].

The hemichannel protein pannexin-1 was identified as contributing to the cationic dye uptake pathway induced by P2X7 activation [7]. However, several studies have brought into question the role of pannexin-1 in the secondary permeability pathway [3], [8], [9]. Schachter *et al* found no pharmacological evidence for pannexin-1 in cationic dye uptake [3] and subsequently the development of a pannexin-1 knockout mouse showed there was no defect in P2X7-induced dye uptake in bone marrow-derived or peritoneal macrophages [8]. Several studies had previously demonstrated an important role for pannexin-1 in P2X7-mediated IL-1β secretion in mouse macrophages [7], [10], [11]. However, Qu *et al* have demonstrated no defect in IL-1β secretion from pannexin-1 deficient macrophages [8]. Similarly there was no defect in transient ATP-induced cell death [9]. Furthermore Alberto *et al* have recently demonstrated a lack of involvement of pannexin-1 in peritoneal murine macrophages [12] casting doubt on the role of this protein in P2X7 mediated signalling.

We are interested in the effect of missense single nucleotide polymorphisms (SNPs) in the human *P2RX7* gene that affect the function and downstream signalling of the ion channel. We recently showed that a gain-of-function SNP encoding an Ala348>Thr mutation in transmembrane domain 2 of human P2X7 is associated with increased inward currents, dye uptake and IL-1β secretion [13]. Our initial aim in this study was to investigate the signalling mechanism linking P2X7 to IL-1β secretion in human monocytes and to understand the contribution of pannexin-1 in this process. We used a range of pharmacological tools to investigate the role of pannexin-1 including carbenoxolone, an inhibitory peptide to pannexin-1 (^10^Panx1), and probenecid. Probenecid is also known to block organic anion transporters [14], but recent studies have demonstrated inhibition of pannexin-1 currents [15], [16]. We found no pharmacological evidence for pannexin-1 involvement in P2X7-mediated dye uptake in HEK-293 cells expressing human P2X7 receptors or in native human monocytes. Conversely probenecid reduced dye uptake in both HEK-293 cells and human monocytes and suppressed ATP-induced IL-1β secretion from human monocytes. Further investigations demonstrated that probenecid reduced P2X7-mediated calcium responses and inward currents in stably transfected HEK-hP2X7 cells suggesting that this compound actually interacts with and directly blocks P2X7.

## Materials and Methods

### Materials

ATP, carbenoxolone and ethidium bromide were from Sigma-Aldrich (St. Louis, MO, USA). Probenecid (water-soluble) and lucifer yellow were from Invitrogen (Carlsbad, CA, USA). AZ11645373, carbenoxolone, AZ10606120, ^10^Panx1 and scrambled peptides were from Tocris Biosciences (Bristol, UK). Ethidium bromide (5 mM) and lucifer yellow (1.6 mg/ml) were prepared in distilled water and stored at 4°C, Probenecid (250 mM) was prepared in distilled water and stored at −30°C. AZ11645373 (50 mM) and AZ10606120 (10 mM) were prepared in DMSO and stocks frozen at −30°C. Carbenoxolone (50 mM) was prepared fresh before each experiment in distilled water or saline buffer. ^10^Panx1 and scrambled peptides (1 mM) were prepared in DMSO and stored at −80°C.

### Cell culture

HEK-293 cells (American Type Culture Collection, Rockville, MD, USA) were cultured as previously described [13]. The HEK-293 human P2X7 stable cell line (HEK-hP2X7) was maintained in complete DMEM∶F12 media containing 10% foetal bovine serum (FBS) (Lonza Australia Pty Ltd, VIC, Australia), 100 U/ml penicillin, 100 μg/ml streptomycin, 5 mM L-glutamine and 800 μg/ml geneticin (all Invitrogen, Carlsbad, CA, USA). Cells were maintained at 37°C in a humidified incubator with 5% CO_2_. The J774 mouse macrophage cell line (American Type Culture Collection, Rockville, MD, USA) was maintained in RPMI-1640 medium supplemented with 10% FCS (Bovogen, East Keilor, Australia) and 2 mM GlutaMax (Invitrogen).

### Isolation of human monocytes

Peripheral venous heparinised blood was obtained from healthy volunteers with informed written consent (approved by Nepean Blue Mountains Local Health Network Human Ethics Committee, 10/40 – AU RED HREC/010/NEPEAN/89). Mononuclear cells were isolated using Ficoll-Paque density gradient centrifugation. CD14 positive monocytes were magnetically selected using CD14 microbeads and MS columns (Miltenyi Biotec, Bergisch Gladbach, Germany) [13].

### Measurement of dye uptake

Dye uptake assays on HEK-hP2X7 cells were performed using a fluorescent plate reader (Optima FLUOSTAR, BMG Labtech). Cells were plated the night before at a density of 5×10^4^ cells/well in 96-well poly-D-lysine coated plates. Ethidium bromide (25 μM) was added to low divalent solution (145 mM NaCl, 5 mM KCl, 0.2 mM CaCl_2_, 13 mM glucose, 10 mM HEPES, pH 7.3). Cells were pre-incubated with drugs for 5–10 minutes at 37°C before measurements started. ATP was injected automatically after 40 seconds. Fluorescence was measured using a 485 nm excitation filter and a 520 nm emission filter block. Gain was set at the beginning of the experiment to 40% required value and fluorescence measurements were taken every 10 seconds for 300 seconds. Data was calculated as baseline corrected endpoint measurements (at 300 seconds) and normalised as percentage of control response.

Ethidium uptake experiments on J774 macrophages were performed using a fixed-time assay as described previously [17] The flow cytometer assay for ATP-induced ethidium uptake in human monocytes was performed as previously described [13]. Briefly, 1×10^6^ peripheral blood mononuclear cells (PBMCs) were stained with APC-conjugated CD14 antibody and FITC-conjugated CD3 antibody (both BD Biosciences) for 15 minutes at room temperature. Cells were washed with PBS and resuspended in 1 ml of low divalent extracellular solution. The tube was warmed to 37°C and 25 μM ethidium bromide added. 1 mM ATP was added after 40 seconds and data acquired every 10 seconds for 400 seconds. Cells were acquired on a BD FACSCalibur flow cytometer using CellQuest software (BD Biosciences, San Jose, CA, USA). Data was calculated as area under the curve (3 minutes) as in previous studies [13].

For lucifer yellow uptake experiments, cells were plated onto 12 mm glass coverslips the day before. Cells on each coverslip were washed briefly in standard extracellular solution (145 mM NaCl, 5 mM KCl, 2 mM CaCl_2_, 1 mM MgCl_2_, 13 mM glucose, 10 mM HEPES, pH 7.3) in a 12-well plate and pre-incubated with probenecid for 10 minutes. Lucifer yellow (LY) was prepared to a final concentration of 0.25 mg/ml in extracellular solution. ATP was added to a final concentration of 3 mM and cells incubated for 15 minutes at 37°C. The coverslip was then washed briefly in standard extracellular solution and transferred into a heated QE-1 chamber mounted on a Nikon Ti-U Eclipse fluorescent microscope. A phase contrast image was taken with a Coolsnap HQ_2_ CCD camera (Photometrics) followed by a fluorescent image using a 380 nm/510 nm filter block and a fixed exposure time of 200 milliseconds. Images were analysed using NIS Elements Advanced Research software version 3.2. Regions of interest (ROIs) were drawn around 50-100 cells and mean intensity calculated.

### Measurement of calcium influx

Cells were loaded with 1 μM Fluo-4AM calcium indicator dye in HBSS buffer containing zero calcium. Loading was performed for 30 minutes at 37°C. Loading buffer was removed and replaced with low divalent assay buffer (145 mM NaCl, 5 mM KCl, 0.2 mM CaCl_2_, 13 mM glucose, 10 mM HEPES, pH 7.3). Cells were pre-incubated with drugs for 5–10 minutes at 26°C before measurements started and ATP was injected automatically after 40 seconds. Fluorescence was measured using a 485 nm excitation and 520 nm emission filter block. Gain was set at the beginning of the experiment to 40% of required value and fluorescence measurements were taken every 10 seconds.

### Electrophysiology

Whole-cell patch-clamp recordings were performed at room temperature (22–26°C) using an EPC10 amplifier and Patchmaster acquisition software (HEKA, Lambrecht, Germany). Agonists and drugs were delivered using the RSC-160 fast-flow system (Bio-Logic Science Instruments, France). Membrane potential was clamped at −60 mV in all experiments. External solution was 145 mM NaCl, 5 mM KCl, 2 mM CaCl_2_, 1 mM MgCl_2_, 13 mM D-glucose, 10 mM HEPES, and internal solution was 145 mM NaCl, 10 mM HEPES, 10 mM EGTA. ATP (1 mM) was applied in low divalent solution. All solutions were adjusted to pH 7.3 with 5 M NaOH and were 300–310 mosm/L.

### IL-1β secretion assays

Magnetically purified CD14^+^ human monocytes were plated at a density of 2×10^5^ cells/well (24-well plate) in RPMI 1640 medium containing 1% FCS as described in [13]. Cells were primed with 100 ng/ml lipopolysaccharide (LPS) for 4 hours and then 3 mM ATP was added for 15 minutes in the presence or absence of pharmacological agents. Cell free supernatants were assayed for IL-1β using a paired antibody ELISA kit (BD Biosciences).

J774 cells were plated at a density of 1×10^6^ cells/well (in 24-well plates) in complete RPMI medium. 1000 ng/ml LPS was used to prime the cells for 4 hours and 5 mM ATP added for 20 minutes in the presence or absence of pharmacological agents. Cell free supernatants were assayed for IL-1β using a murine IL-1β ELISA Max Deluxe kit (Biolegend, San Diego, CA, USA).

### Statistical analysis

Data are reported as mean ± SEM or standard deviation from three to four separate experiments. Graphs and statistical analysis was performed using GraphPad Prism 6 (GraphPad Software Inc., San Diego, CA, USA) and Student's t-test or one-way ANOVA were used for statistical significance where P<0.05.

## Results

### No evidence for pannexin-1 involvement in P2X7 receptor -induced dye uptake

**Figure 1:**
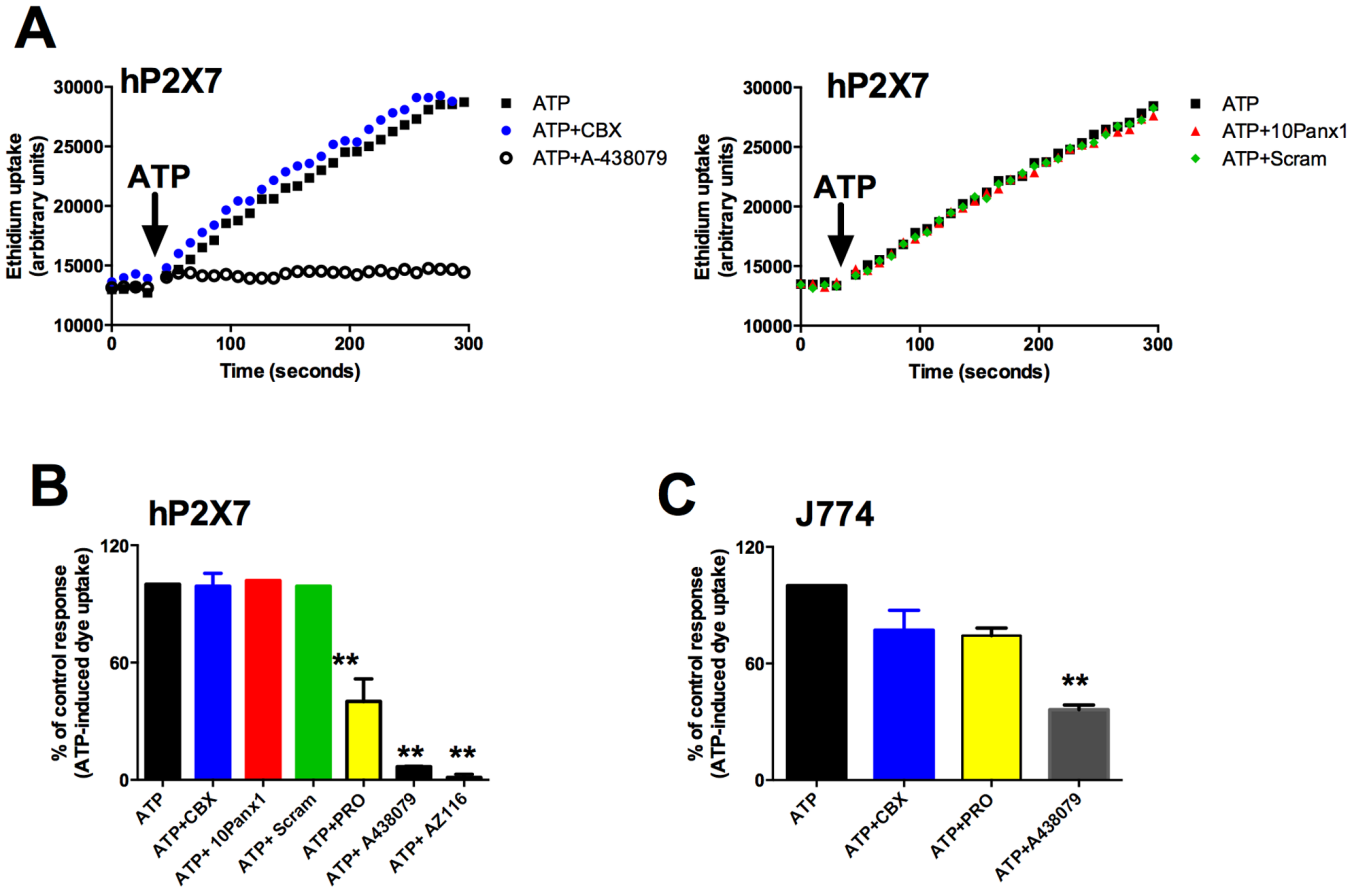
Effect of pannexin-1 inhibitors on ATP-induced dye uptake in HEK-293 cells expressing human P2X7 and J774 macrophages. (A) Ethidium^+^ uptake was induced in HEK-hP2X7 expressing cells by the addition of 1 mM ATP (denoted by the arrow) in low divalent physiological solution. Control ethidium uptake to ATP is shown in black squares. Cells were pre-incubated with inhibitors for 10 minutes at 37°C before ATP addition. In the top panel ethidium uptake in the presence of 10 μM carbenoxolone (CBX) is shown in blue circles and in the presence of 10 μM A438079 in black open circles. In the lower panel ethidium uptakes in the presence of the pannexin-1 peptide (^10^Panx1) or scrambled ^10^Panx1 peptide are shown in red triangles or green diamonds respectively. Cellular fluorescence was measured using a fluorescent plate reader over 300 seconds. (B) Bar chart displaying mean data from 2–5 separate experiments using inhibitors carbenoxolone (CBX), ^10^Panx1 peptide or scrambled ^10^panx1 peptide (100 μM), A-438079 and AZ11645373. Data was calculated at 300 second timepoint as baseline corrected endpoint data and normalised to the ATP control. (C) ATP (3.4 mM) induced ethidium uptake was measured in J774 macrophages. CBX (50 μM), PRO (2.5 mM) and A438079 (10 μM) significantly reduced the response. Error bars represent S.E.M, ** P<0.05 calculated by one-way ANOVA (Dunnett's post-test).

We studied P2X7-induced dye uptake in HEK-293 cells using ethidium bromide (final concentration 25 μM) and a fluorescent plate reader. P2X7 was activated by 1 mM ATP in a low divalent (200 μM CaCl_2_, zero MgCl) sodium chloride buffer. We tested a range of drugs previously shown to act on pannexin-1 [15] – carbenoxolone (CBX), an inhibitory peptide to pannexin-1 (^10^Panx) and probenecid (PRO). In HEK-293 cells stably expressing the human P2X7 receptor (HEK-hP2X7), concentrations up to 100 μM CBX had no effect on ATP-induced dye uptake (Figure 1) whereas the selective P2X7 blockers A-438079 and AZ11645373 showed complete blockade of ATP-induced dye uptake in HEK-293 cells at 10 μM (Figure 1B). The ^10^Panx1 peptide also had no effect on ATP-induced dye uptake in HEK-hP2X7 (Figure 1). Probenecid showed a significant inhibitory effect at 2.5 mM, reducing ATP-induced dye uptake to 40±5.1% of control (n = 5, P<0.01). Data is summarised in Figure 1B. In contrast to HEK-hP2X7 cells, ATP-induced dye uptake was reduced by both CBX and probenecid in J774 macrophages (Figure 1C) and both drugs could reduce spontaneous dye uptake in HEK-293 cells transfected with pannexin-1 (Figure S1).

### Probenecid blocks P2X7-induced dye uptake in HEK-hP2X7 cells and human monocytes

**Figure 2:**
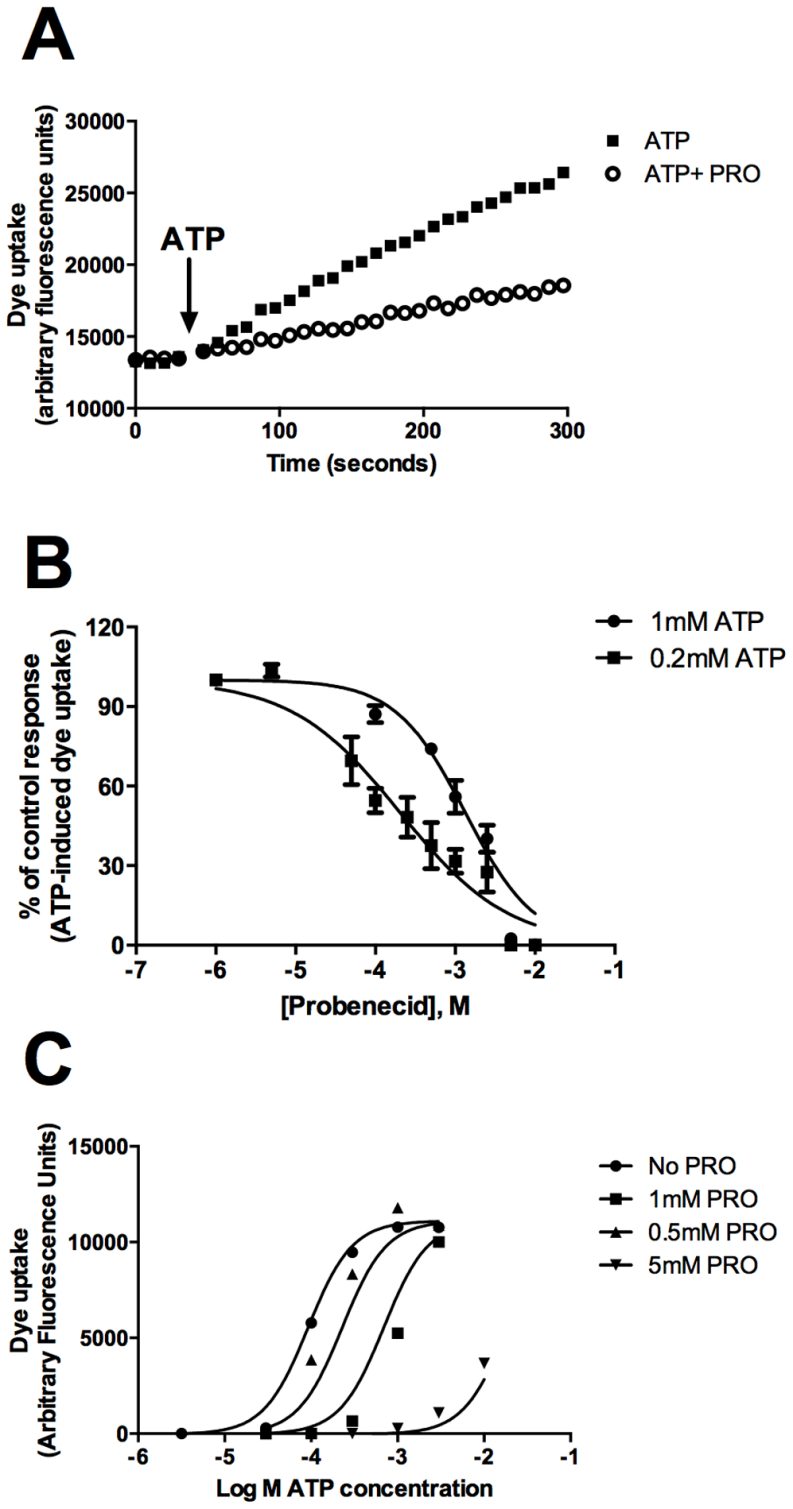
Probenecid inhibits ATP-induced dye uptake in HEK-hP2X7 cells. (A) Ethidium^+^ uptake was induced in HEK-hP2X7 expressing cells by the addition of 1 mM ATP (as denoted by the arrow) in low divalent physiological solution. Control ethidium uptake to ATP is shown in black squares and ethidium uptake in the presence of 2.5 mM probenecid (pre-incubated for 10 minutes at 37°C) is shown in open circles. (B) Concentration response curves for probenecid on human P2X7 dye uptake responses induced by 0.2 mM or 1 mM ATP. Data was calculated at 300 second timepoint as baseline corrected endpoint data and normalised to the ATP control. Data was fit using a normalised response variable slope in GraphPad Prism. (C) Concentration response curves for agonist ATP in the presence of increasing concentrations of probenecid (0.5 mM, 1 mM and 5 mM) showing a rightward shift.

**Figure 3:**
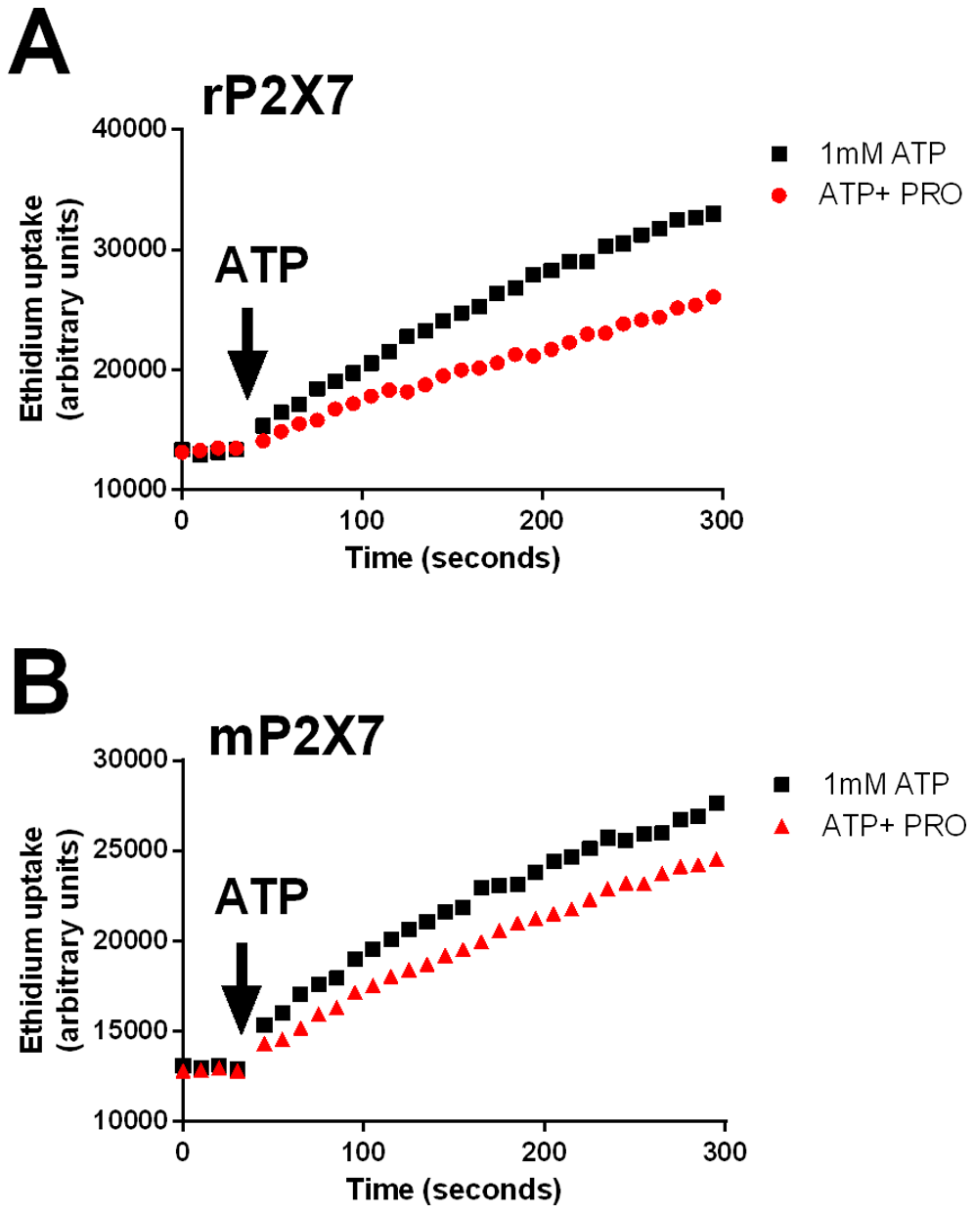
Probenecid shows minimal inhibition of ATP-induced dye uptake in HEK-rat P2X7 and HEK-mouse P2X7 cells. Ethidium^+^ uptake was induced in (A) rat P2X7 and (B) mouse P2X7 stable cell lines by the addition of 1 mM ATP (denoted by the arrow) in a low divalent physiological solution. Control ethidium uptake to ATP is shown in black squares and ethidium uptake in the presence of 2.5 mM probenecid (pre-incubated for 10 minutes at 37°C) is shown in red shapes.

**Figure 4:**
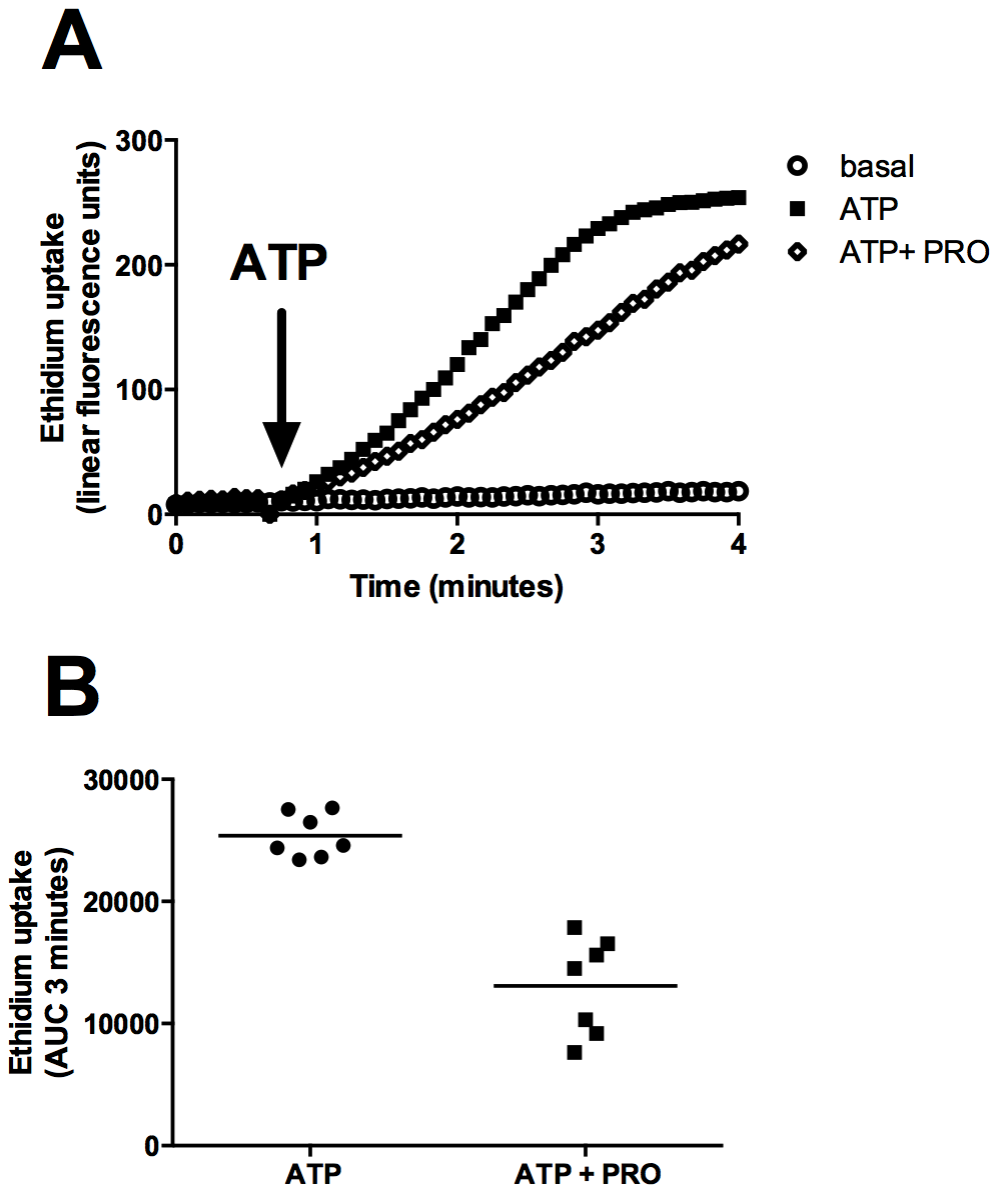
Probenecid inhibits P2X7-induced dye uptake in human CD14^+^ monocytes. Ethidium^+^ uptake was induced in CD14-APC labelled human monocytes by the addition of 1 mM ATP (denoted by the arrow) in low divalent KCl buffer in the absence and presence of 1 mM probenecid. Dye uptake was measured on a FACSCalibur flow cytometer using a heated time-resolved module. A representative uptake curve is shown from 6 donors (top panel). Data (area under the curve after 3 minutes) from all donors in shown in bottom panel scatter plot.

We further investigated the inhibitory effect of probenecid in HEK-hP2X7 cells (Figure 2A) and constructed a concentration-response curve. Probenecid blocked P2X7-mediated dye uptake with an IC_50_ value of 203 μM (95% CI 138–298 μM) when using 0.2 mM ATP as agonist (Figure 2B). Raising the concentration of ATP to 1 mM increased the IC_50_ value of probenecid to 1.3 mM (95% CI 1.0–1.7 mM) (Figure 2B). A full concentration response for the P2X7 agonist ATP was then performed in the absence and presence of increasing concentrations of probenecid (0.5 mM, 1 mM and 5 mM) to determine if probenecid acts in a competitive manner. A rightward shift in the dose response curve was observed in the presence of probenecid indicating that probenecid may be a competitive inhibitor of human P2X7 (Figure 2C). We next tested whether probenecid would also block rat P2X7 and mouse P2X7 expressed in HEK-293 cells. Probenecid (2.5 mM) also blocked ATP-induced dye uptake in the rat P2X7 stable cell line (34% inhibition) and to a lesser extent in the mouse P2X7 stable cell line (20% inhibition) (Figure 3). We then determined if this inhibitory effect of probenecid was similar in P2X7-induced dye uptake response in human monocytes. Probenecid (1 mM) reduced dye uptake in human monocytes to 51% of the control ATP response in human CD14^+^ monocytes (Figure 4B, n = 7 donors). As observed for HEK-hP2X7, CBX and ^10^Panx1 peptide failed to impair ATP-induced dye uptake into human monocytes whereas the P2X7 antagonist A-438079 (10 μM) blocked dye uptake completely in these cells (Figure S2).

**Figure 5:**
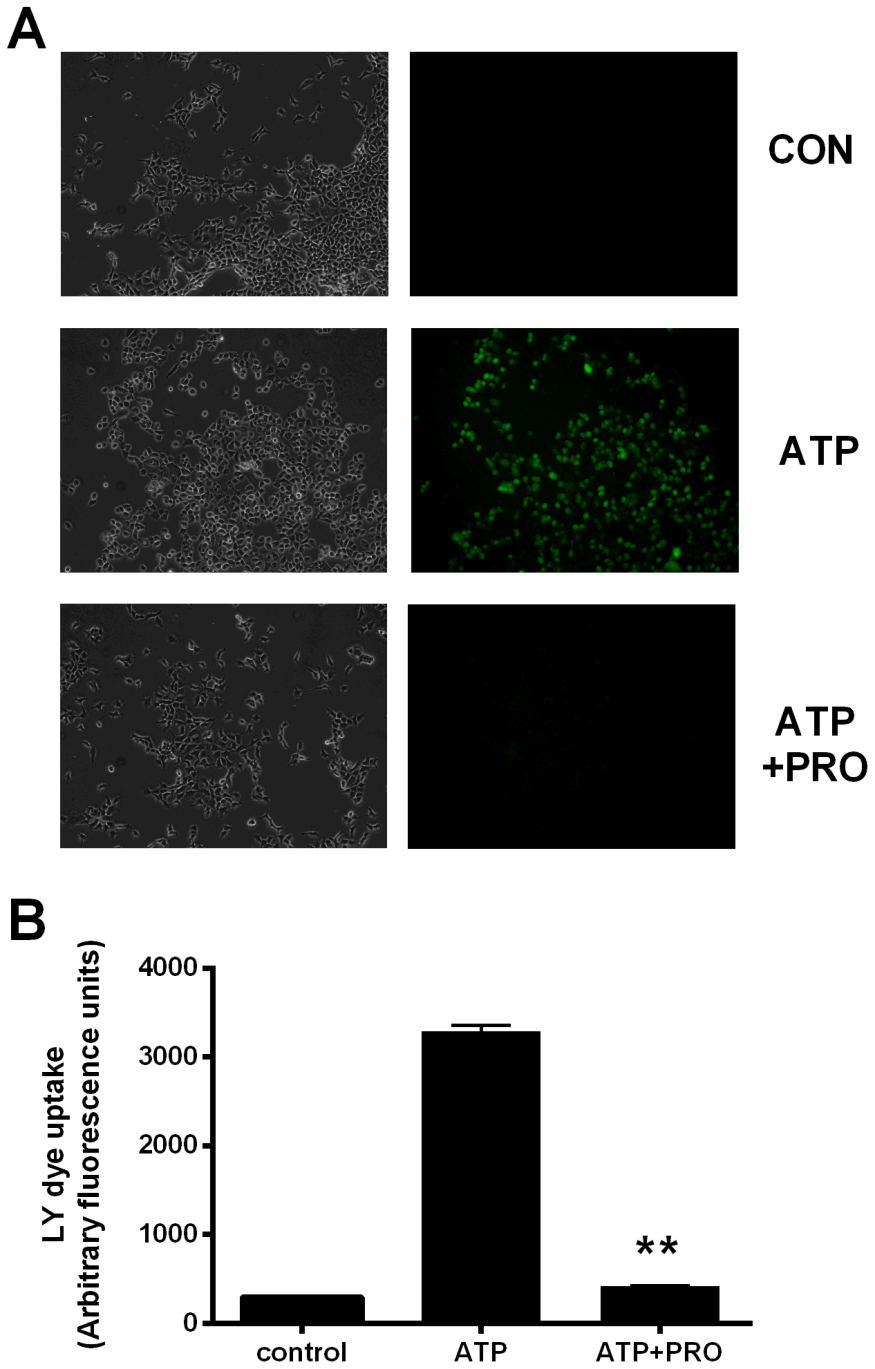
Probenecid blocks lucifer yellow uptake in HEK-hP2X7 cells. Lucifer yellow (0.25 mg/ml) uptake in response to P2X7 stimulation by ATP (3 mM) was measured using a fixed time incubation at 37°C. Coverslips were briefly washed in standard extracellular solution before being imaged. (A) Phase contrast and fluorescent images for each stimulation condition; Control, ATP (3 mM) and ATP+PRO (2.5 mM) and (B) mean data from 100–300 cells (regions of interest) from multiple images.

To further investigate the effect of probenecid on dye uptake induced by P2X7, we determined whether anionic dye uptake was also inhibited by this compound. We measured ATP-induced LY uptake in HEK-hP2X7 cells and found that probenecid could significantly block LY uptake (Figure 5) in addition to cationic dye uptake (Figure 1, 2). This also demonstrates that probenecid blocks P2X7 signalling in standard extracellular solutions containing both calcium and magnesium.

### Probenecid but not CBX reduces P2X7-dependent IL-1β secretion from monocytes

**Figure 6:**
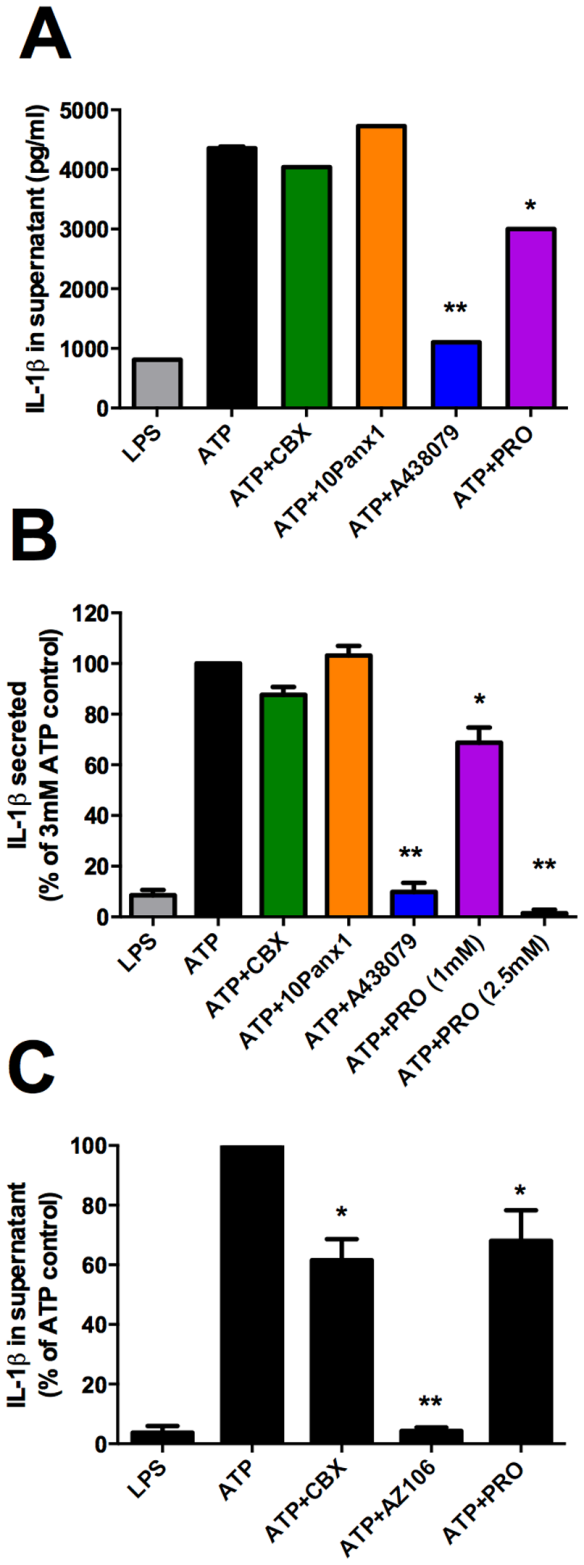
Probenecid but not pannexin-1 blockers reduce P2X7-induced IL-1β secretion from human monocytes and J774 macrophages. Magnetically isolated CD14^+^ human monocytes were primed with LPS (100 ng/ml) for 4 hours and stimulated with 3 mM ATP for 15 minutes. After stimulation supernatants were removed and tested for IL-1β. (A) representative data from one donor, (B) shows pooled data from 6 donors expressed as % of ATP control. Carbenoxolone (CBX) was used at 50 μM, probenecid (PRO) at 1 mM and 2.5 mM, ^10^Panx1 at 100 μM, and A-438079 at 10 μM. (C) The J774 mouse macrophage cell line was primed with LPS (1 μg/ml) and stimulated with ATP (5 mM) for 20 minutes in physiological saline. P2X7 selective antagonist AZ10606120 (AZ106) was used at 10 μM. Mean data from three independent experiments is shown. ATP-induced IL-1β released into supernatant was measured by specific ELISAs for human and mouse IL-1β respectively. Error bars represent S.E.M and * represents P<0.05.

We next determined whether pannexin-1 inhibitors and probenecid could affect P2X7-dependent IL-1β secretion from lipopolysaccharide (LPS) primed CD14^+^ primary human monocytes. As seen with dye uptake (Figure 4, Figure S2) CBX and ^10^Panx1 peptide had no effect on ATP-induced IL-1β secretion from human monocytes (Figure 6) whereas A438079 completely blocked ATP-induced IL-1β secretion. Figure 6A shows the effect of CBX (50 μM), ^10^panx1 peptide (100 μM), A-438079 (10 μM) and probenecid (1 mM) from a representative experiment (one donor) and Figure 6B shows average normalised data from six separate donors. A-438079 significantly blocked ATP-induced IL-1β secretion to 9.9±8% of the ATP control, a similar level of secretion as LPS alone (8.5±5.3% of ATP control, n = 6 donors). CBX (87.7±6% of control) and ^10^Panx1 (103.1±9.4% of ATP control) had no significant effect. Probenecid (1 mM) reduced ATP-induced IL-1β secretion to 68±14.6% of control (n = 6 donors, P<0.05, one-way ANOVA with Dunnett's post-test) and a concentration of 2.5 mM probenecid reduced ATP-induced IL-1β secretion to 4.3% of control (n = 3 donors, P<0.05, one-way ANOVA with Dunnett's post-test). As previously observed by others [7] we confirmed that CBX could impair ATP-induced IL-1β secretion from J774 macrophages (Figure 6C). In addition, the P2X7 antagonist AZ1061620 (10 μM) and probenecid (2.5 mM) also showed a significant reduction in ATP-induced IL-1β secretion (n = 3 experiments, P<0.05, one-way ANOVA with Dunnett's post-test).

### Probenecid blocks ATP-induced calcium influx and inward currents in HEK-hP2X7 cells

**Figure 7:**
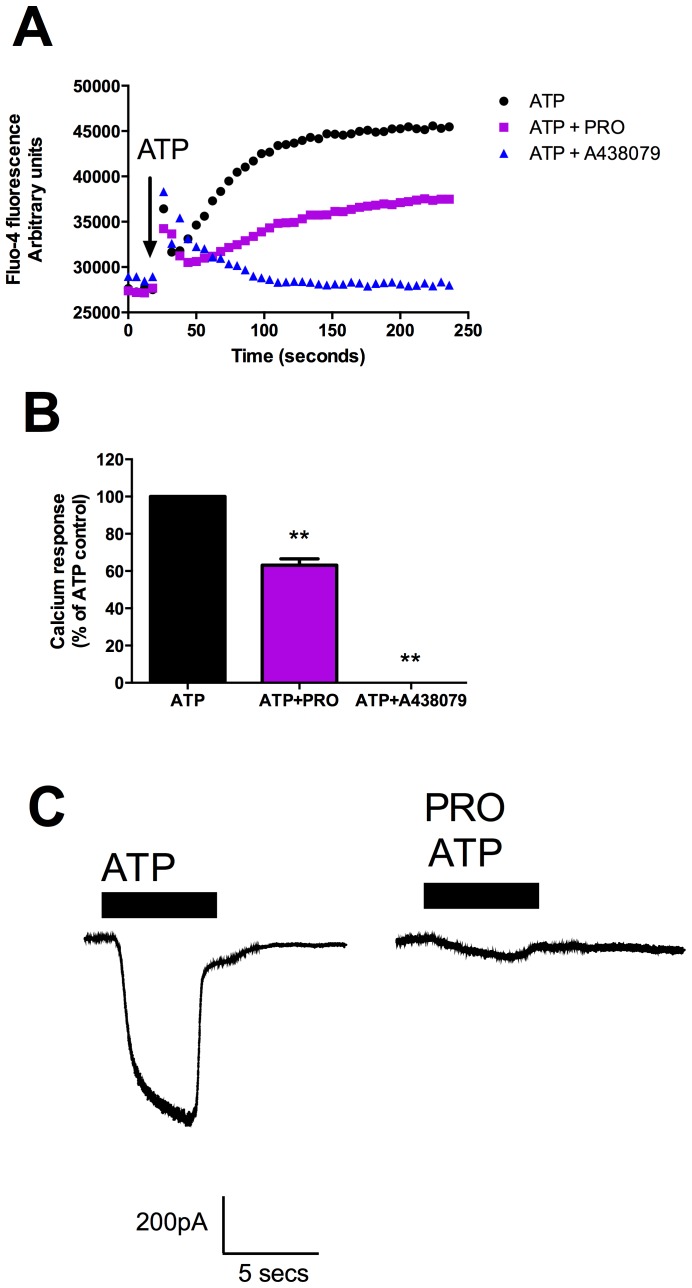
Probenecid blocks P2X7-mediated calcium influx and inward currents in HEK-hP2X7 cells. HEK-hP2X7 cells were loaded with 1 μM Fluo-4 for 30 minutes and calcium responses recorded at room temperature (26°C) using a fluorescent plate reader. ATP (1 mM) was injected and fluorescence recorded at 520 nm in the absence of inhibitors or in the presence of 1 mM probenecid (purple) or 10 μM A-438079 (blue). (B) Mean data from three independent calcium experiments. Sustained portion of the calcium response was measured and calculated as % of ATP control. Probenecid reduced response to 63±3.4% of control whereas A-438079 completely abolished the sustained calcium response. (C) Inward currents through wild-type human P2X7 receptors expressed in HEK-293 cells were recorded using whole cell patch clamp at room temperature. Membrane was clamped at −60 mV and ATP (1 mM in low divalent solution) was applied using a fast-flow delivery system. Black bars indicate ATP exposure (5 seconds). The initial ATP response was measured then the cell was exposed to probenecid for 2 minutes before re-challenge with ATP in the continued presence of probenecid.

To determine if probenecid was blocking P2X7 directly as suggested in Figure 2 we measured signalling events occurring before pore dilation, namely channel opening. P2X7 is a non-selective cation channel with high calcium permeability and will induce a sustained calcium influx response to ATP. Probenecid is widely used in loading and retaining calcium indicator dyes in the cytoplasm of cells [18]. HEK-hP2X7 cells were loaded with Fluo-4AM calcium indicator dye and calcium responses were recorded at 26°C to reduce Fluo-4 extrusion through the probenecid-inhibitable transporter. A large sustained calcium response was measured following the addition of 1 mM ATP that was completely blocked by the presence of 10 μM A-438079 (Figure 7A). Probenecid (1 mM) reduced this sustained calcium response to 63±5.8% of control (Figure 7A) supporting the idea that probenecid is blocking P2X7 directly. To confirm this we recorded inward currents through P2X7 stably expressed in HEK-293 cells using whole-cell patch clamp. A pulse of ATP (1 mM) was applied for 5 seconds using a fast-flow agonist delivery system and cells were bathed in standard extracellular saline solution containing 1 mM probenecid for 2 minutes before re-application of ATP. The resulting ATP-induced inward currents were significantly reduced in presence of probenecid (Figure 7C); mean current density to ATP was 70.9±7.2 pA/pF and with ATP + probenecid 27.9±3.9 pA/pF (n = 5 cells, p = 0.0024, students t-test).

## Discussion

Probenecid has previously been shown to block the pannexin-1 hemichannel [15], [16] in addition to inorganic anion transporters [14], [19], TASR16 [20], and unusually has agonist activity at TRPA1 and TRPV2 channels [21], [22]. It is also a commonly used laboratory agent to enhance cellular retention of fluorescent calcium indicator dyes [18]. Here we demonstrate for the first time that probenecid also has a direct inhibitory effect on the human P2X7 receptor. Our data reveal human P2X7 as another target for probenecid and we suggest that caution be used when using this compound to block pannexin-1 particularly in human cells. Our data also indicates that measuring P2X7-dependent calcium influx in buffers containing probenecid will underestimate such responses and other methods should be used to retain indicator dyes.

Consistent with other studies we find no pharmacological evidence for pannexin-1 being involved in the P2X7 mediated secondary permeability pathway [3], [8], [12]. There are limited selective tools for pannexin-1; carbenoxolone (CBX) is routinely used as a pannexin-1 antagonist in the concentration range 1–20 μM [15] and a pannexin-1 mimetic peptide (^10^Panx1) [7] is probably the most selective tool available to block pannexin-1 responses. We found no inhibitory effect on ATP-induced dye uptake in HEK-hP2X7 cells using CBX and ^10^Panx1 whereas P2X7 selective antagonists were extremely effective in blocking the P2X7 dye uptake response (Figure 1). We also found no effect of CBX and ^10^Panx1 on ATP-mediated dye uptake or IL-1β secretion from native human monocytes (Figures 4, 6). Moreover, we have recently observed that probenecid but not CBX blocks ATP-mediated dye uptake in myeloid leukaemic cells [23] however the mechanism of probenecid action was not established. In contrast to our studies with human cells, CBX and probenecid did reduce ATP-mediated dye uptake and IL-1β secretion in J774 mouse macrophages as previously observed [7], [12] suggesting a role for pannexin-1 in this macrophage cell line.

Probenecid was the only pannexin-1 antagonist to show marked inhibition of ATP-induced dye uptake in our HEK-hP2X7 stable cell line, reducing the response by 60% in our screening experiments. We determined an IC_50_ value of 203 μM (95% confidence interval 138–298 μM) blocking responses elicited by 200 μM ATP on human P2X7. Using a higher ATP concentration to stimulate P2X7 increased the IC_50_ value to 1.3 mM (95% confidence interval 1.0–1.7 mM). This suggested that probenecid may compete with ATP at the agonist binding site. Further dose response experiments suggested that probenecid is a competitive inhibitor of P2X7 due to the rightward shift in EC_50_ for the agonist ATP (Figure 2C). The calculated IC_50_ value for probenecid on human P2X7 is similar to that reported for the urate transporter [24] and is within the therapeutic range for uricosuric activity of probenecid (100–200 μg/ml). To further demonstrate that probenecid is likely binding to P2X7 directly rather than blocking the secondary permeability pathway (which could be mediated by other (unknown) proteins), we measured calcium influx and inward currents through the P2X7 ion channel. Probenecid effectively reduced calcium influx through the P2X7 channel as measured by the calcium indicator dye Fluo-4 and blocked inward currents through the P2X7 channel as measured using whole cell patch clamp (Figure 7). Therefore, probenecid can be added to a growing list of compounds with effect at P2X7 including the protein kinase C inhibitor chelerythrine [25], a tyrosine kinase inhibitor [26], MAP kinase inhibitors such as SB203580 [27] and the phospholipase D inhibitor CAY10593 [28]. Probenecid also reduced spontaneous dye uptake in HEK-293 cells transfected with pannexin-1 (Figure S1) and therefore we suggest that probenecid can block pannexin-1 as previously demonstrated [15], [16] and additionally it can block human P2X7.

Probenecid could also block anionic dye uptake into HEK-hP2X7 cells (Figure 5). These experiments confirm findings by others [3] that HEK-293 cells expressing P2X7 can mediate uptake of the anionic dye lucifer yellow in a calcium and magnesium containing extracellular solution.

One major implication of inhibition of human P2X7 by probenecid is the underestimation of calcium influx in fluorescent calcium indicator dye experiments. Many protocols and kits include probenecid at millimolar concentrations to prevent the transporter mediated efflux of dyes such as Fluo-4 and Fura-2 out of the cytosol during the loading period [29]. Therefore any investigation of P2X7 responses particularly in human primary cells or cell lines would not accurately measure P2X7-mediated calcium influx. Alternative compounds such as sulfinpyrazone could be used, although our preliminary experiments suggest that this may also inhibit P2X7 responses (data not shown). Therefore in order to measure P2X7 mediated calcium flux in human cells it is necessary to eliminate the use of dye loading enhancers such as probenecid. In our experience this can be achieved by loading cells with the Fluo-4AM ester at 37°C for 30–45 minutes followed by removal of the Fluo-4AM containing buffer. Fresh assay buffer is added to the cells and the measurement of intracellular calcium signals is then performed at 26°C to minimise dye extrusion. Our experiments in Figure 7 utilised this particular protocol with measurable P2X7 calcium responses. While the P2X7 cationic pore pathway is known to be sensitive to changes in temperature with reduced responses at temperatures below ambient room temperature (20–25°C) [3], in our experiments ATP-induced cationic dye uptake in HEK-293 cells was similar at 26°C and 37°C (data not shown).

Probenecid is a uricosuric agent clinically used to reduce serum urate levels in the autoinflammatory arthritic disease gout [30]. Individuals with high serum uric acid levels deposit uric acid crystals in joints which activate acute inflammatory reactions leading to swelling and pain [31]. Studies have shown that uric acid crystals activate the NLRP3 inflammasome in joint resident macrophages causing pro-inflammatory cytokine secretion and further recruitment of immune cells [32], [33]. The major cytokine implicated in the pathogenic inflammation associated with gout is IL-1β [34]. P2X7 plays a key role in regulating activation of NLRP3 inflammasome by the danger signal ATP and the induction of rapid secretion of IL-1β from monocytes and macrophages [4]. Recent studies suggest that uric acid crystals activate the NLRP3 inflammasome by releasing ATP from monocytes or macrophages [33], [35]. Reduction of signalling through P2X7 may be a beneficial off-target effect of probenecid in gout. Colchicine is a second gout treatment recently shown to block ATP-induced dye uptake in cells expressing P2X2 or P2X7 receptors [36]. Colchicine was also demonstrated to reduce ATP-induced IL-1β secretion from macrophages [36]. Future investigations into the role of P2X7 in gout may be worthwhile [37].

## Supporting Information

Figure S1 **CBX and PRO reduce spontaneous YO-PRO uptake in HEK-293 cells transfected with pannexin-1.** YO-PRO iodide uptake was induced in HEK-293 cells expressing pannexin-1 by injection of the dye (40 seconds) to a final concentration of 5 μM. Cells were incubated in buffer alone (red), 100 μM CBX (blue) or 2.5 mM PRO (purple) for 10 minutes before dye injection. Fluorescence was measured using a Fluostar Optima plate reader and graph is representative of several experiments.
(TIF)

Figure S2 **Pannexin-1 antagonists have no inhibitory effect on ATP-induced dye uptake in human monocytes.** Ethidium^+^ uptake was induced in CD14-APC labelled human monocytes by the addition of 1 mM ATP (denoted by the arrow) in low divalent KCl buffer in the absence and presence of 50 μM CBX (blue, top panel), 100 μM ^10^Panx1 (red, middle panel), or 10 μM A-438079 (open circles, bottom panel). Dye uptake was measured on a FACSCalibur flow cytometer using a heated time-resolved module. A representative uptake curve is shown from several donors.
(TIF)
