# Supplementary figures and images for: Probenecid Blocks Human P2X7 Receptor-Induced Dye Uptake via a Pannexin-1 Independent Mechanism
Source: PLoS One. 2014 Mar 26;9(3):e93058. doi: 10.1371/journal.pone.0093058 (PMC3966854; doi:10.1371/journal.pone.0093058)

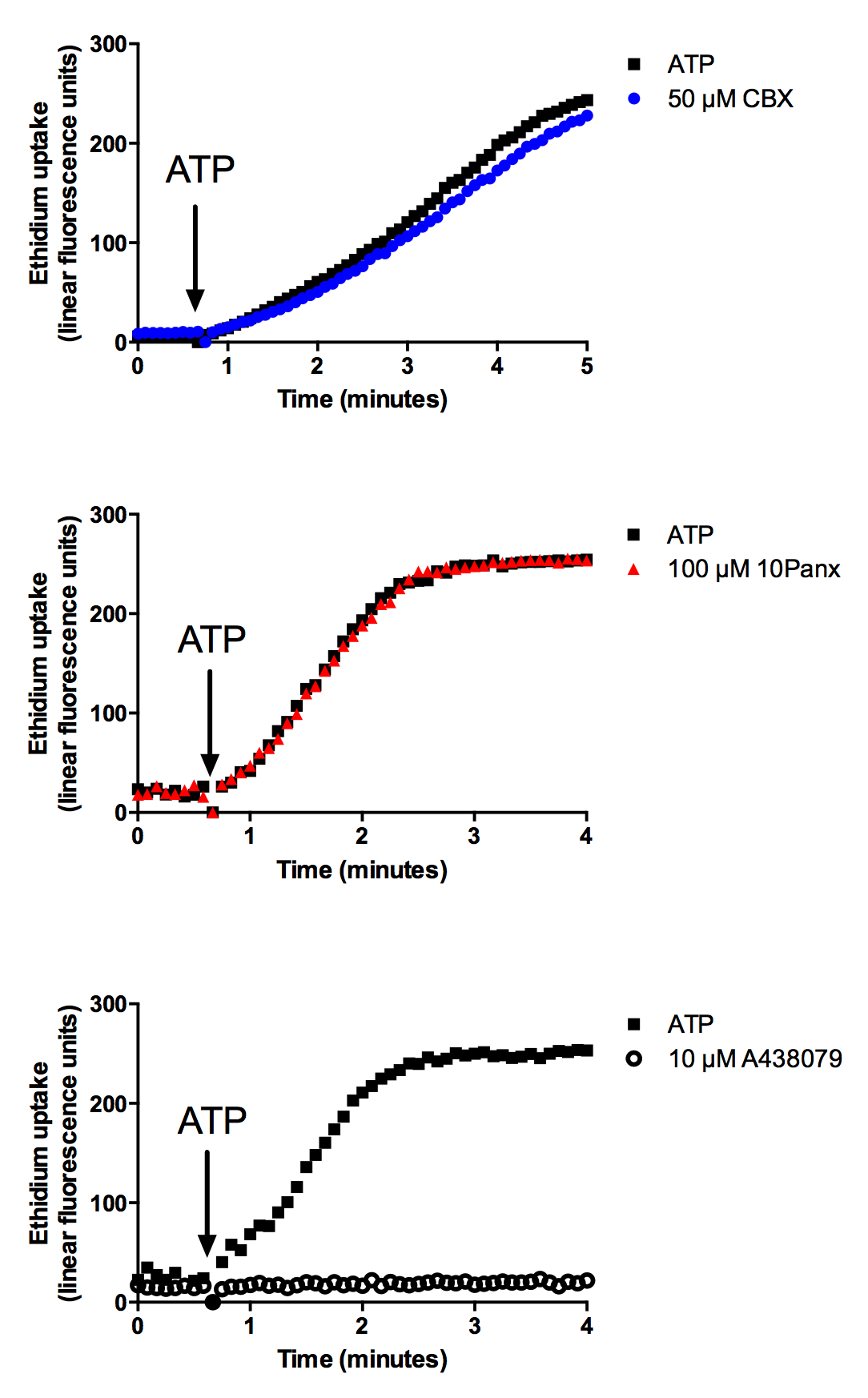

Supplement: Figure S2 — Pannexin-1 antagonists have no inhibitory effect on ATP-induced dye uptake in human monocytes. Ethidium+ uptake was induced in CD14-APC labelled human monocytes by the addition of 1 mM ATP (denoted by the arrow) in low divalent KCl buffer in the absence and presence of 50 μM CBX (blue, top panel), 100 μM 10Panx1 (red, middle panel), or 10 μM A-438079 (open circles, bottom panel). Dye uptake was measured on a FACSCalibur flow cytometer using a heated time-resolved module. A representative uptake curve is shown from several donors. (TIF) [file pone.0093058.s002.tif]
